# Research Advances and Detection Methodologies for Microbe-Derived Acetylcholinesterase Inhibitors: A Systemic Review

**DOI:** 10.3390/molecules22010176

**Published:** 2017-01-23

**Authors:** Jingqian Su, Huiying Liu, Kai Guo, Long Chen, Minhe Yang, Qi Chen

**Affiliations:** 1 Fujian Key Laboratory of Innate Immune Biology, Fujian Normal University, Fuzhou 350117, China; sjq027@fjnu.edu.cn (J.S.); liubrusc@163.com (H.L.); gk9309@yeah.net (K.G.); 2 Biomedical Research Center of South China, Fujian Normal University, Fuzhou 350117, China; 3 College of Life Science, Fujian Normal University, Fuzhou 350117, China; minhe214@fjnu.edu.cn; 4 Tumor Invasion Microecological Laboratory, the First Affiliated Hospital of Fujian Medical University, Fuzhou 350005, China; charmed1905@163.com

**Keywords:** Alzheimer’s disease, acetylcholinesterase inhibitors, in vitro assays

## Abstract

Acetylcholinesterase inhibitors (AChEIs) are an attractive research subject owing to their potential applications in the treatment of neurodegenerative diseases. Fungi and bacteria are major producers of AChEIs. Their active ingredients of fermentation products include alkaloids, terpenoids, phenylpropanoids, and steroids. A variety of in vitro acetylcholinesterase inhibitor assays have been developed and used to measure the activity of acetylcholinesterases, including modified Ellman’s method, thin layer chromatography bioautography, and the combined liquid chromatography-mass spectrometry/modified Ellman’s method. In this review, we provide an overview of the different detection methodologies, the microbe-derived AChEIs, and their producing strains.

Acetylcholinesterase is a secretory carboxylesterase present in the central and peripheral nervous systems. This enzyme rapidly hydrolyze the neurotransmitter acetylcholine into choline and ultimately terminates the excitatory effects of acetylcholine on the postsynaptic membrane, ensuring the proper transduction of nervous signals and critically affecting the cholinergic nervous system [1]. In addition to presenting at the neuromuscular junctions, the cholinergic system is also widely expressed in the cortex, basal ganglia, hypothalamus, brainstem nuclei, and cerebellum, and has functions in attention, memory, execution, language, and other cognitive processes [2]. In addition to its role in transducing nervous signals, acetylcholinesterase is also involved in the other cellular processes, such as cell differentiation, apoptosis, and cell adhesion [3].

Acetylcholinesterase inhibitors (AChEIs) extend and enhance the effects of acetylcholine by causing the acetylcholine accumulation in the synapses. AChEIs are usually made by chemical synthesis or produced by plants and microorganisms, and may have applications in treating neurodegenerative disorders such as Alzheimer’s disease.

In this review, we illustrate a variety of microorganism-derived AChEIs and summarized recent advances in the detection assays used for testing AChEI activity.

## 1. Effects of AChEIs 

AChEIs can be categorized as strong or weak inhibitors. Strong inhibitors include organic phosphates and carbamates, which are primarily used as neural toxins and pesticides, while weak inhibitors have been used in the treatments of Alzheimer’s disease (AD), dementia with Lewy bodies, Parkinson’s disease, myasthenia gravis, glaucoma, postural tachycardia syndrome, autism, insomnia, and cognitive disorders [4]. Currently, AChEIs are most often studied in the context of their applications in the treatment of AD and myasthenia gravis. AD is a common degenerative disease of the central nervous system in the elderly, with clinical manifestations including memory decline and impaired social recognition [5,6]. To date, several AChEIs have been used in clinical practice for treating AD, including donepezil, rivastigmine, galanthamine, and huperzine A, among which galanthamine and huperzine A are natural compounds [7].

Myasthenia gravis is a chronic autoimmune disease resulting from dysfunction of the acetylcholine receptor at the neuromuscular junction. The symptoms of myasthenia gravis include weakness or abnormalities in the involved skeletal muscles and susceptibility to fatigue, which may increase after intense activities. Symptoms can be partially alleviated after rest or by the use of cholinesterase inhibitors, such as pyridostigmine and neostigmine [8].

## 2. In Vitro Assays for AChEIs

Most original in vitro assays for testing AChEI activity are conducted by using preparations of isolated guinea pig ileum smooth muscles. These assays involve complicated protocols, including a long preparation time, and the use of large amounts of animal tissues and reagents, which constrains the development of large-scale screening and detection of AChEIs. These assays have been replaced by biochemical assays that are more sensitive and less complicated [9], which will be elucidated in the following.

### 2.1. Ellman’s Method

The chemical principle of Ellman’s method involves hydrolysis of acetylcholine by acetylcholinesterase to produce acetic acid and choline, which then reacts with Ellman’s reagent (5,5′-bisdithionitrobenzoic acid [DTNB]) to form a compound exhibiting light absorbance at 412 nm. With other factors unchanged, the absorbance value is linearly correlated with the inhibition activity of the enzyme, facilitating calculation of the half-maximal inhibitor concentration (IC50) [10]. Although Ellman’s method does not require the preparation of animal tissues for in vitro analysis of AChEIs, it is restricted to a small pH range (6.5–8.5), in which the disulfide bond breakage in DTNB occurs, thereby limiting the assay for use within this pH range. The –SH group, which detaches from the protein, can also react with DTNB and interfere with the hydrolysis reaction; however, the results are often not ideal and therefore cannot be used in high-throughput screens. 

To overcome these problems, Gorun et al. [11] made various modifications to the assay as follows. Instead of adding the DTNB reagent and substrates into the reaction system at the same time, DTNB is added into the solution after the incubation of acetylcholinesterase and substrates, followed by termination and color development. In this context, the enzymatic reaction and the chromogenic step which are affected by the pH are separated; these modifications permit the detection of enzyme activity at any pH and thereby increase the assay sensitivity. The use of a microplate/microplate reader also allows for further simplification of Ellman’s method, thereby significantly decreasing the experimental duration [12,13]. Bajda et al. [14] developed the electrophoretically mediated microanalysis (EMMA) technique for rapid screening of AChEIs. The data obtained from the EMMA assay and Ellman’s test had good linear relationships in the range of their respective mass concentration. Ignasik et al. [15] used both methods for testing AChE inhibitory activities of a series of new 2-(diethylaminoalkyl)-isoindoline-1,3-dione derivatives designed using molecular modeling. Both methods gave a satisfactory result, which lends support for EMMA as a reliable tool for rapid screening of novel cholinesterase inhibitors.

### 2.2. Thin-Layer Chromatography (TLC) Bioautography

One of the disadvantages of the modified Ellman’s method in screening for AChEIs is the higher frequency of false-positive results, which stems from the inhibitory activities of acetylcholinesterase by residues of organic solvents used in sample preparation and purification. In addition, the production cost increases with the number of isolation and purification steps increases. Therefore, an important tactic is to reduce the number of steps during isolation and purification, as well as to reduce the interference from organic solvents [16]. Rhee et al. combined TLC with the modified Ellman’s method and developed a TLC bioautography method to effectively solve the above problems. In the procedure, test samples are effectively separated by silica gel TLC. After the organic solvents are completely evaporated, the chromogenic reagent DTNB is first sprayed on the chromatography plates, followed by spraying with acetylcholinesterase. After acetylcholinesterase reacts with the sample thoroughly, the acetylcholine substrate is sprayed on the plate. The appearance of white spots in the orange or yellow background indicates the possibility of the existence of compounds with acetylcholinesterase inhibition activity, and further separation and purification can be conducted [17].

To further eliminate the possible errors caused by the naked, Marston et al. used acetylcholinesterase to decompose naphthyl acetate into naphthol, which can react with Fast Blue B to form a lustrous purple diazo compound [18]. The distinction of white spots in a purple background has a better visual effect and increases the sensitivity up to 1 × 10^−10^ μg/L. Yang et al. [19] optimized the use of acetylcholinesterase, substrate (naphthyl acetate), and chromogenic agent (Fast Blue B), which leads to an 85% reduction in acetylcholinesterase usage while adhering to the same sensitivity of 1 × 10^−10^ μg/L. Ramallo et al. [20] screened for AChEIs by combining TLC bioautography with high-resolution mass spectrometry (HRMS). In comparison to direct mass spectrometric analysis, the combined system is more affordable and exhibits improved detection specificity.

### 2.3. Combining Liquid Chromatography-Mass Spectrometry (LC-MS) with the Modified Ellman’s Method

Ingkaninan et al. [21] modified the Ellman’s method by synchronously coupling liquid chromatography-mass spectrometry (LC-MS) which allows simultaneous separation, purification, and structural analysis of the sample, as well as detection of AchEI activity. The coupled LC-MS/modified Ellman’s method system requires three reagents: DNTB, AChE, and acetylthiocholine iodide (ATCI). The detection instrument therefore also needs to be equipped with three respective samplers, which causes the inevitable problems in synchronizing the ultraviolet spectrum, mass spectrometry, and biochemical detection in the system. Furthermore, the chromogenic reagent DNTB can result in chromogenic reaction with impurities in the sample, leading to false-positive results. However, despite these problems, this detection system is still advantageous and shows high selectivity and sensitivity. For example, for galanthamine, the detection sensitivity for this method can reach 0.3 nM. To resolve the above problems, Rhee et al. (2003b) [22] have used a synthetic nonfluorescent substrate, 7-acetoxy-1-methyl quinolinium iodide (AMQI), which can be decomposed into a strong fluorescent reagent, 7-hydroxy-1-methyl quinolinium iodide (HMQI), in the improved system, therefore the AChEI activity can be determined by fluorescence intensity. Based on the example of galanthamine, multiple compounds with AChEI activity were isolated and screened from plants of the Amaryllidaceae family. This method only requires one reagent to be added, thus reduces the quantity of reagents used. However, the nonfluorescent substrate AMQI is only stable at low pH and is susceptible to self-decomposition under the optimal pH range, which makes it difficult to detect the actual inhibitory capacity of the compounds being tested.

De Jong et al. [23] made further improvements to the experimental system, in which the test samples were separated by high-performance liquid chromatography (HPLC), followed by reacting with acetylcholinesterase and the substrate acetylcholine. Then, mass spectrometry is used to determine the quantity of acetylcholine and choline contents, and the sample’s AchEI activity is then obtained. The studies have shown that this system is simple and stable.

Kukula-Koch et al. [24] have used a combined system utilizing countercurrent chromatography, TLC bioautography, liquid chromatography, and time-of-flight (TOF) mass spectrometry to first separate components of root extracts from *Argemone mexicana* L. by countercurrent chromatography, followed by TLC autography to determine the bioactivity, and finally HPLC-electrospray ionization (ESI)-TOF-MS for purification to obtain molecular information of compounds. Using this study, compounds such as berberine, protopine, chelerithrine, sanguinarine, coptisine, palmatine, magnoflorine, and galanthamine have been identified. Additionally, galanthamine was found for the first time in *Argemone mexicana* L.

Different methods of in vitro AChEI detection have their specific advantages and disadvantages (Table 1), and current research predominantly employs the modified Ellman’s method and TLC bioautography; further modifications to these procedures should be made according to particular experimental conditions.

## 3. Types of AChEIs Produced by Microbes

The isolation of AChEIs has progressed from initial extraction from plants to the screening of AchEI-producing strains from soil and isolation from cyanobacteria (initially thought to be dead green algae floating on top of stagnant water), lichens, and endophytic fungi within plants. The products mainly include alkaloids, terpenoids, and other compounds. As shown in Table 2, as of 2015, a total of 31 strains have shown to contain compounds with AChEI activity in their fermentation products. Except for one strain of lichen (*Cladonia macilenta Hoffm*), the 36 remaining strains are fungi and bacteria, including 12 *Deuteromycotina*, 11 *Ascomycota*, one Cyanophyta, one Basidiomycota, and 11 bacteria, primarily Actinomycetes and Cyanophyta. The homogeneity of these strains could provide insights into future screening of AchEI producers.

The active ingredients of fermentation products mainly include alkaloids (50% of all active ingredients), phenylpropanoids, terpenoids, and steroids. The active ingredients of two strains have not been successfully isolated. Out of the 12 *Deuteromycotina* strains, seven produce alkaloids (six of which produce huperzine A), four produce terpenoids or steroids, one produces multiple types of active ingredients, and one produces unknown active ingredients. Out of the 11 Ascomycotina strains, five produce alkaloids (three of which produce huperzine A), four produce phenylpropanoid derivatives, and two produce terpenoid derivatives. Out of 11 strains of bacteria, six produce alkaloids (two of which produce huperzine A), two produce phenylpropanoid derivatives, two produce other types of compounds, and one produces unknown active ingredients.

### 3.1. AChEIs from Fungi

As shown in Table 2, 20 AchEI producing fungi include *Aspergillus terreus*, *Aspergillus terreus* (No. GX7-3B), *Acremonium implicatum* LF30, *Acremonium* sp. 2F09P03B, *Aspergillus flavus* LF40, *Botrytis* sp. HA23, *Cladosporium cladosporioides* LF70, *Paecilomyces tenuis* YS-13, *Alternaria* sp. YD-01, *Aspergillus flavus*, *Phomopsis* sp., *Aspergillus versicolor* Y10, *Trichoderma* sp., *Chrysosporium* sp., *Penicillium citrinum*, *Penicillium* sp. EPF-6, *Penicillium* sp. FO-4259, *Penicillium* sp. sk5GW1L, *Penicillium chermesinum* ZH4-E2, *Xylaria* sp., *Hyalodendriella* sp. Ponipodef12, *Penicillium griseofulvum* LF146, *Shiraia* sp. Slf14, and *Xylaria* sp. YS-02. These fungi belong to the *Deuteromycotina* and *Ascomycota* phyla subdivisions, among which five stains belong to *Penicillium*, accounting for 25% of the studied strains (Table 2).

#### 3.1.1. Alkaloid AChEIs and the Producing Fungi

Huperzine A is the most studied alkaloid AchEI originating from fungi. The nine strains shown to produce huperzine A by fermentation account for 39% of all strains studied (Table 2). Huperzine A possesses a multitude of pharmacological effects. In addition to being a highly selective and efficient AchEI in the central nervous system, huperzine A can also provide protection for cells by inhibiting excitotoxicity induced by glutamate and by blocking oxidative stress and apoptosis. Additionally, huperzine A may exert therapeutic effects in a variety of degenerative diseases, including myasthenia gravis, memory disorders, vascular dementia, mental retardation in children due to iodine deficiency, and chronic insomnia. 

In recent years, several research groups in China have isolated and screened several huperzine A-producing endophytic fungal strains from Huperziaceae and determined the product quantities of each strain [27,35,36,37,38,40,44,45,61,62,63]. The productivity of most strains is less than 60 μg/L, with *Shiraia* sp. Slf14 being the best producer (327.8 μg/L of the fermentation product) [44]. The optimal culturing conditions and molecular properties have been set for this strain [27,44,64,65,66].

Li et al. [37] optimized the fermentation conditions for huperzine A production in endophytic fungi through both one-factor and orthogonal tests. Additionally, based on a model system two huperzine A-producing endophytic fungal strains, *Xylaria* sp. YS-02 and *Paecilonmyces tennis* YS-13, with production levels of 26.4 and 21.0 μg/L, were identified, respectively, through screening 127 isolated endophytic fungus strains in three Huperziaceae (*Phlegmariurus austrosinicus*, *Phlegmariurus petiolatus*, and *Huperzia serrata*). The two strains producing huperzine A by fermentation were reported for the first time.

Kakinuma et al. [31] isolated the strain *Penicillium* sp. EPF-6, which produces quinolactacins A, B, and C, from the larvae of *Diaphania pyloalis* (Lepidoptera: Pyralidae) for the first time. These three types of quinolactacins share a unique quinolone backbone and a common chromophore (C_12_H_8_N_2_O_2_). The quinolactacins A showed inhibitory activity on TNF production by murine macrophages and macrophage-like J774.1 cells stimulated with LPS. The mixture of quinolactacins A and B, as well as racemate quinolactacin C, inhibited the yeast α-glucosidase in a concentration-dependent fashion with IC_50_ values of 273.3 µM and 57.3 µM [67].

Kim et al. [30] isolated quinolactacins A1 and A2 from the fermentation solid of *Penicillium citrinum*. Structural analysis revealed that the chemical structure of quinolactacin A1 is identical to that of quinolactacin A and that the structure of quinolactacin A2 is identical to that of quinolactacin B. Quinolactacin A1 is a diastereomer of quinolactacin A2. Kim et al. [30] used Ellman’s method to detectrmine the AchEI activity of quinolactacins A1 and A2 and showed that quinolactacin A2 exhibited stronger AchEI activity (IC_50_ = 19.8 μM). Based on the “OSMAC” (one strain-many compounds) technique, Teles et al. [68] isolated and purified paecilomide, a pyridone alkaloid, from *Paecilomyces lilacinus* using a combination of the modified Ellman’s method and TLC bioautography. The acetylcholinesterase inhibition efficiency of paecilomide is 57.5% ± 5.50% at the dose of 10 mg/mL.

#### 3.1.2. Terpenoid AChEIs and the Producing Fungi

**Figure 1:**
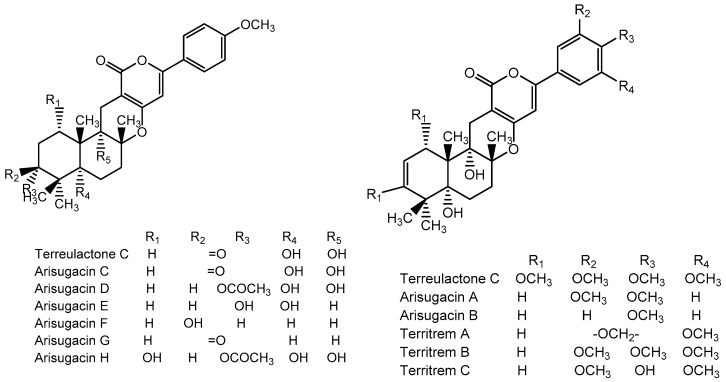
Structures of the arisugacins A–H, territrems A–C, and terreulactones C and D.

Terpenoid AChEIs extracted from fungi are mostly diterpenoids and meroterpenoids, respectively. Ling et al. [25] isolated a strain of *Aspergillus terreus* from unhusked rice (paddy) and obtained three diterpenoid compounds, i.e., territrem A, territrem B, and territrem C, from the fermentation products (Figure 1). Peng et al. [69] confirmed that five derivatives of territrem B possess the AchEI activity. Using AchEI assays, Peng et al. [69] confirmed that territrem B is able to inhibit acetylcholinesterase (IC_50_ = 7.6 μM).

As early as 1995, researchers at Taiwan University initiated screens for microbe-originated AchEI, and Omura et al. [32], in Japan, reported a series of Arisugacins compounds (Figure 1) that effectively inhibit the acetylcholinesterase activity. Arisugacins are shown to be produced by *Penicillium* sp. FO-4259 isolated from soil in the Minato-ku region of Tokyo, Japan. Arisugacins A and B share the same backbone with the territrems reported by Ling et al. (1984) [70]. This implies that the same backbone of these compounds has much effect in mediating their anti-AChE activity. Moreover, Huang et al. [33] extracted a strain of *Penicillium* sp., sk5GW1L, from Kandelia candel, from which three terpenoid compounds, i.e., arigsugacin I, arigsugacin F, and territrem B (Figure 1), were isolated and purified; these compounds exhibited prominent AchEI activity, with IC_50_ values of 0.64, 0.37, and 7.03 μM, respectively.

**Figure 2:**
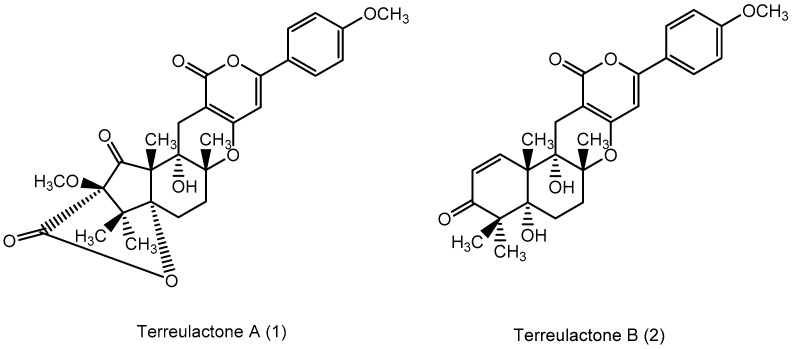
Structures of the terreulactones A and B.

**Figure 3:**
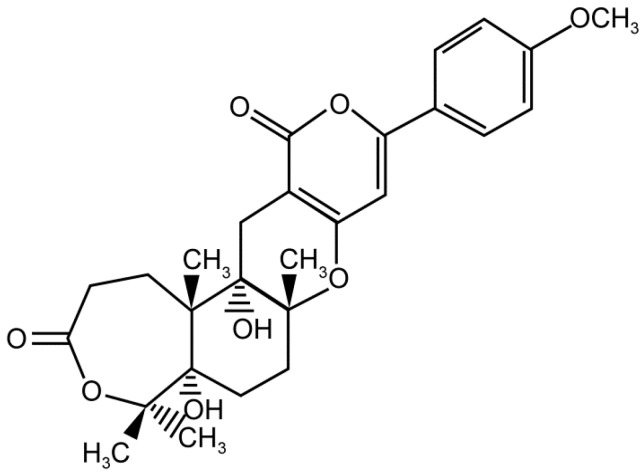
Structure of isoterreulactone A.

From fermentation products of a *Aspergillus terreus* strain isolated from soil, Cho et al. [71] were able to isolate and purify four polyterpenoid compounds with polyketide structures (terreulactones A and B Figure 2) that show the AChEI activity. Terreulactone A is a meroterpenoid with a sesquiterpene lactone structure consisting of a sesquiterpene portion and a lactone backbone; the IC_50_ of terreulactone A is 0.23 μM. In 2005, Yoo et al. [72] also reported a new meroterpenoid (isoterreulactone A; Figure 3) isolated from the fermentation products of *Aspergillus terreus*, with an IC_50_ of 2.5 μM. Terreulactone C shares the same backbone with arisugacins C–H and has an IC_50_ of 0.23 μM. Terreulactone D shares the same backbone as arisugacins A and B, and territrems A–C, and its IC_50_ is 0.42 μM. In 2014, Kim et al. [73] obtained a strain of *Streptomyces* sp. from screens, and anithiactins A–C were isolated and purified from the metabolites. All three compounds were shown to have moderate AChEI effects, and the IC_50_ for these three compounds are 63, 53, and 68 μM. The anithiactins A–C did not show any significant cytotoxicity against A-498 and ACHN human cancer cell, at concentrations of lines up to 200 μM with a modified MTT assay. The analysis of correlation of acetylcholinesterase activity and chemical structure indicated that α-Pyrone merosesquiterpenoids possessing an angular tetracyclic carbon skeleton are frequently isolated from the genera *Penicillium* and *Aspergillus* as anti-cholinesterase active constituents [74].

#### 3.1.3. Other Types of AChEIs from Fungi

Marine microorganisms which can be produced in a large quantity have also become a nature source used for isolating AChEIs. Lin et al. [46] and Luo et al. [75] isolated a strain of *Xylaria* sp. from mangrove; five structurally similar oxygen heterocyclic compounds xyloketals A–E were further isolated from the fermentation broth. The xyloketals A–E have a highly substituted core aromatic ring and one or more ketals. Using the modified Ellman’s method, xyloketals A–D are shown the AChEI activity in vitro, with IC_50_ values of 29.9, 137.4, 109.3, and 425.6 μM. Analysis of enzyme kinetic curves has indicated that xyloketals A–D are a type of highly selective, noncompetitive, reversible AChEIs. In atherosclerotic apoE^−/−^ mice, xyloketal B attenuated the atherosclerotic lesion area, which showed neuroprotective and scavenged DPPH free radical activities [76]. Liu et al. [77] isolated 43 strains of marine fungi from seabed sediments in Lianyungang, China. From them, the ethyl acetate extracts were made with Ellman’s method. Of these 43 strains, 15 extracts display 50% AChEI activity and 3 extracts (L1705, S1101 and SH0701) with more than 80% AChEI activity at the concentration of 500 mg/mL, with IC_50_ values of 11.3 ± 1.2, 72.1 ± 2.3 and 7.8 ± 2.8 mg/mL, respectively. Particularly, the fungus SH0701 was identified as *Aspergillus ochraceus* SH0701. After polarity gradient fractionation, the results showed that the ethyl acetate fraction of *Aspergillus ochraceus* SH0701 possessed the highest AChEI activity with an inhibition rate of 71.55% at a concentration of 10 mg/mL. Thus, the above results revealed that marine microorganisms could be another important source of novel AChEIs.

Additionally, researchers have isolated fungi from soil and plants, and obtained a multitude of compounds with the AChEI activity from fermentation products. For example, Rao et al. [43] reported the isolation of a strain of *Chrysosporium* sp. from the fermentation broth, from which an AchEI (14-(2′,3′,5′-trihydroxyphenyl)tetradecan-2-ol) was extracted.

**Figure 4:**
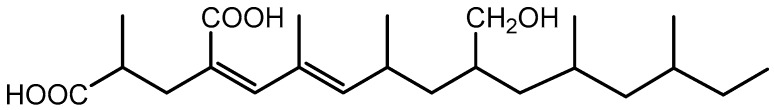
Structure of the compound F99-909A.

Notably, as a aromatic alkyl compound, 14-(2′,3′,5′-trihydroxyphenyl)tetradecan-2-ol was shown to have an IC_50_ of 231 μM as measured by a modified method of Ellman’s. Moreover, Zheng et al. [78] isolated a fungal strain from the soil of Yunnan, China. The fermentation products of this strain were shown to contain an AchEI (F99-909A; Figure 4) with an IC_50_ of 20 μM using the modified Ellman’s method. Chemical structure analysis revealed that this compound has a spatial structure identical to that of radclonic acid, an active compound with stimulatory effects on plant growth.

Meng et al. (2012) [47] and Mao et al. [48] isolated an endophytic fungal strain *Hyalodendriella* sp. Ponipodef12 from *Populus cathayana* and screened its metabolites to obtain four benzopyran ketone compounds: palmariol B, 4-hydroxymellein, alternariol 9-methyl ether, and botrallin. Using the modified Ellman’s method, these compounds were shown to have IC_50_ values of 115.31, 116.05, 135.52, and 83.70 µg/mL. This was the first report on the antinematodal and AChEI activities of palmariol B, 4-hydroxymellein and alternariol 9-methyl ether. The experimental results showed that palmariol B had stronger antimicrobial, antinematodal and AChEI activities than alternariol 9-methyl ether, which indicated that the chlorine substitution at position 2 may contribute to its bioactivity. As the same endophytic fungus *Hyalodendriella* sp. Ponipodef12, Mao et al. [48] described the isolation and applied high-speed counter-current chromatography (HSCCC) and semi-preparative HPLC technology for preparative separation of dibenzo-a-pyrones from the fungus for the first time.

Chapla et al. [42,79] screened and isolated two endophytic fungal strains *Phomopsis* sp. and *Colletotrichum gloeosporioides* from *Senna spectabilis* and *Michelia champaca*, respectively. Seven compounds, including cytochalasin H, were isolated and purified from the fermentation broth of *Phomopsis*. Cytochalasin H has strong inhibitory activity against acetylcholinesterase in vitro, as shown by TLC bioautography. The results suggested that *C. gloeosporioides* plays an important ecological role in protecting *M. champaca* against phytopathogens. The potent antifungal activity of the novel natural product cytochalasin H, which demonstrated activity against pathogenic fungi, suggested that the endophyte and the host plant can produce substances that show antifungal activity against possible phytopathogenic fungi or that are harmful to predators of the plant.

Wang et al. [29] isolated *Aspergillus versicolor* Y10 from *Huperzia serrata* and purified five compounds from its fermentation products. Using huperzine A as the positive reference (IC_50_ = 0.6 μM), the AchEI IC_50_ of the purified compound avertoxin B was shown to be 14.9 μM with the modified Ellman’s method.

### 3.2. AChEIs from Bacteria

From the 1980s to the 1990s, while the world was focusing on the development of plant and chemical synthetic AChEIs, Murao et al. [52] isolated a physostigmine-producing strain of *Streptomyces* sp. AH-14 from Sakai’s soil in Japan for the first time. Additionally, Ohlendorf et al. [53] obtained a new phenazine-derived natural product, geranylphenazinediol, from the fermentation products of *Streptomyces* sp. isolated from the seabed sediment. This compound shows strong AchEI activity with an IC_50_ value of 2.62 μM when analyzed using the modified Ellman’s method.

Kurokawa et al. [54] reported the isolation of an oxygen heterocycle compound, cyclophostin, from *Streptomyces lavendulae*. In vitro activity assays demonstrated that cyclophostin possesses high AchEI activity, with an IC_50_ of 7.6 μM as measured by the modified Ellman’s method. Additionally, from the metabolites of Actinobacillus N98-1021, Dong et al. [56] isolated the compound N98-1021A, which exhibits the AchEI activity using the modified Ellman’s method and shows structural similarity to terferol. Moreover, Pandey et al. [50] extracted a *Bacillus subtilis* strain-M18SP4P from *Fasciospongia cavernosa* and showed that this strain blocks 54% of acetylcholinesterase activity. Using galanthamine as a positive reference, TLC bioautography identified two components in the metabolites of M18SP4P with the AchEI activity. This was the first report on the AChE inhibition activity in the microbial associates of the marine invertebrates and sediment. Li et al. [55] isolated and purified two compounds from the marine actinomycete *Rubrobacter radiotolerans*. Both of these compounds show moderate AchEI activity as measured by the modified Ellman’s method, with IC_50_ values of 11.8 and 13.5 μM, respectively. Wu et al. [58] obtained nine compounds from the fermentation broth and mycelia of a *Talaromyces* sp. strain LF458. Of these nine compounds, talaromycesone A, talaroxanthenone, and AS-186c showed moderate inhibitory activities using the modified Ellman’s method, with IC_50_ values of 7.49, 1.61, and 2.60 μM, respectively. This study first demonstrated the AchEI activity of oxaphenalenone.

### 3.3. AChEIs from Other Species

Additional AchEI-producing species include *Cladonia macilenta* and *Haddowia longipes*. Luo et al. [59] isolated and purified a rare natural phenanthrenequinone compound (biruloquinone) from the fermentation broth of *Cladonia macilenta*. Biruloquinone has strong AchEI activity as measured by the modified Ellman’s method, with an IC_50_ of 27.1 μg/mL. From the ethyl acetate extracts of *Haddowia longipes*, Zhang et al. [49] isolated nine lanosteroids and nine known compounds. Using tacrine as a positive reference, 100 μM test samples were prepared from each compound. Of the 18 compounds, 13 exhibited the AchEI activity, with inhibition efficiencies between 10.3% and 42.1%, which was assayed by the spectrophotometric method developed by Ellman. The inhibition efficiency of tacrine is 65.8%. In coincidence with the structure-activity analyses, the compound 4 among these lanostanoids displayed stronger AChEI activities than compound 5, which suggest an important role of the methylation of hydroxyl group substituted in C-25 of the lanostanoid backbone. At the same time, compound 8 had significantly higher activity than compounds 16 and 17, which indicating that the hydroxyl group at C-15 was required and seemed more important for activity comparing the hexa-nor lanostanoids with the same skeleton.

## 4 Conclusions

As the clinical demand for AChEIs continues to increase, in vitro assays for AChEIs are also undergoing rapid development, particularly with the development of Ellman’s method, which allows for in vitro high-throughput screening. The development of the modified Ellman’s method, TLC bioautography, combined LC-MS and modified Ellman’s method, and other methods has significantly improved isolation and screening techniques for AchEI-producing microorganisms, which predominantly consist of fungi and bacteria. The AchEI-producing fungi are mainly *Ascomycotina* and *Deuteromycotina*, whereas the primary AchEI-producing bacteria include *Actinobacteria* and *Cyanophyta*. AchEI produced by fungi include alkaloids, terpenoids, phenylpropanoids, and steroids. Current research continues to focus on the isolation and screening of producing strains, and few reports have described strain modification, product yield increases, metabolic pathways, and genetic engineering. Thus, these areas still need to be further explored. Additionally, further improvements of in vitro AchEI detection methods may be achieved through construction of molecular models, a combination of bioinformatics simulation and biological analysis to predict the amino acids within the active site of acetylcholinesterase, and analysis of the functional mechanisms of the tested compounds.

## Figures and Tables

**Table 1:** Comparison of methods used for in vitro AChEI detection.

Method	Advantages	Disadvantages
Ellman’s method	In vitro testing; no requirement for animal tissues	Large consumption of reagents; difficulty in large-scale preparation of some samples; often nonideal results, inappropriate for high-throughput screens
Modified Ellman’s method	Simplified assay protocol, reduced experimental time, high-throughput screens	Complicated protocol for isolation and purification of samples; interference from organic solvents
TLC bioautography	Reduced steps in sample isolation and purification protocol; elimination of interference from organic solvents	False-positive experimental results
Combined LC-MS/modified Ellman’s method	High specificity and detection sensitivity	Synchronous coupling by the instrument, requirement of additional samplers; false-positive experimental results; expensive materials

**Table 2:** Microbial producers of AChEIs.

Strain	Classification	Structure of Active Ingredient(s)	Compound Type	IC_50_	Method Used	Ref.
*Aspergillus terreus*	*Eumycota*, *Deuteromycotina*, *Hyphomycetes*, *Moniliales*, *Moniliaceae*, *Aspergillus*	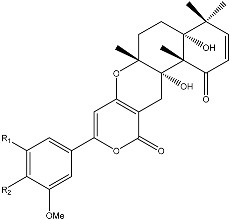	Territrem B	7.6 μM	Ellman’s method	Ling et al. [25]
*Aspergillus terreus* (No. GX7-3B)	*Eumycota*, *Deuteromycotina*, *Hyphomycetes*, *Moniliales*, *Moniliaceae*, *Aspergillus*	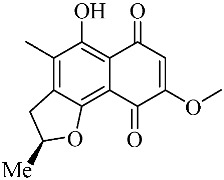	Anhydrojavanicin	2.01 μM	Modified Ellman’s method	Deng et al. [26]
		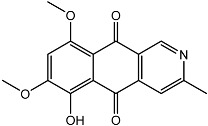	8-*O*-Methylbostrycoidin	6.71 μM		
		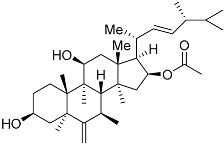	NGA0187 (Terpenoid)	1.89 μM		
		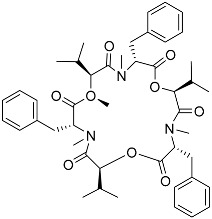	Beauvericin	3.09 μM		
*Aspergillus flavus* LF40	*Eumycota*, *Deuteromycotina*, *Hyphomycetes*, *Moniliales*, *Moniliaceae*, *Aspergillus*	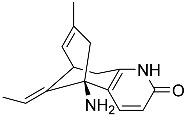	Huperzine A	0.6 μM	Modified Ellman’s method	Wang et al. [27]
*Aspergillus flavus*	*Eumycota*, *Deuteromycotina*, *Hyphomycetes*, *Moniliales*, *Moniliaceae*, *Aspergillus*	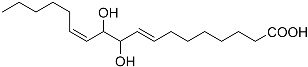	(8*E*,12*Z*)-10,11-Dihydroxyoctadeca-8,12-dienoic acid	No report	Modified Ellman’s method	Qiao et al. [28]
		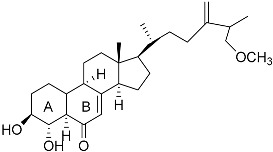	3β,4α-Dihydroxy-26-methoxyergosta-7,24(28)-dien-6-one			
*Aspergillus versicolor* Y10	*Eumycota*, *Deuteromycotina*, *Hyphomycetes*, *Moniliales*, *Moniliaceae*, *Aspergillus*	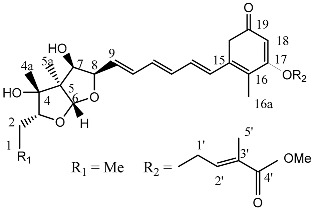	Avertoxin B	14.9 μM	Modified Ellman’s method	Wang et al. [29]
*Penicillium citrinum*	*Eumycota*, *Deuteromycotina*, *Hyphomycetes*, *Moniliales*, *Moniliaceae*, *Penicillium*	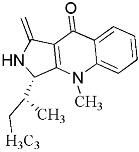	Quinolactacins A1	280 μM	Modified Ellman’s method	Kim et al. [30]
		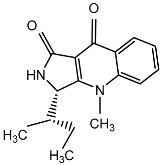	Quinolactacins A2	19.8 μM		
*Penicillium* sp. EPF-6	*Eumycota*, *Deuteromycotina*, *Hyphomycetes*, *Moniliales*, *Moniliaceae*, *Penicillium*	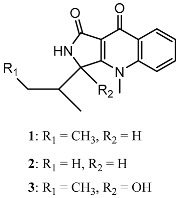	Quinolactacins A, B, and C	No report	Ellman’s method	Kakinuma et al. [31]
*Penicillium* sp. FO-4259	*Eumycota*, *Deuteromycotina*, *Hyphomycetes*, *Moniliales*, *Moniliaceae*, *Penicillium*	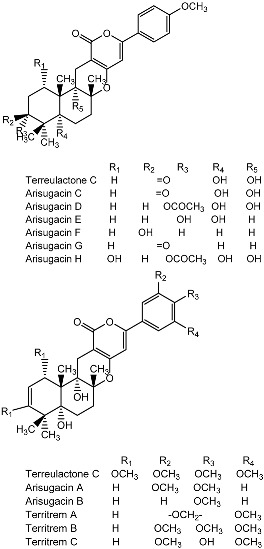	Arigsugacin I	0.64 μM	Modified Ellman’s method	Omura et al.; Huang et al. [32,33]
*Penicillium* sp. sk5GW1L	*Eumycota*, *Deuteromycotina*, *Hyphomycetes*, *Moniliales*, *Moniliaceae*, *Penicillium*					
			Arigsugacins F	0.37 μM		
			Territrem B	7.03 μM		
*Penicillium chermesinum* (ZH4-E2)	*Eumycota*, *Deuteromycotina*, *Hyphomycetes*, *Moniliales*, *Moniliaceae*, *Penicillium*	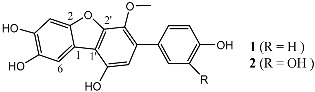	Terphenyls	7.8 μM 5.2 μM	Modified Ellman’s method	Huang et al. [34]
*Penicillium griseofulvum* LF146	*Eumycota*, *Deuteromycotina*, *Hyphomycetes*, *Moniliales*, *Moniliaceae*, *Penicillium*	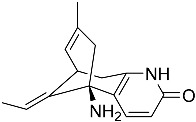	Huperzine A	0.6 μM	Modified Ellman’s method	Wang et al. [35]
*Paecilomyces tenuis* YS-13	*Eumycota*, *Deuteromycotina*, *Hyphomycetes*, *Paecilomyces Paecilomyces*	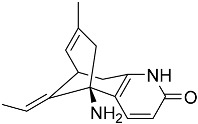	Huperzine A	0.6 μM	Modified Ellman’s method	Su and Yang [36]
*Acremonium implicatum* LF30	*Eumycota*, *Deuteromycotina*, *Hyphomycetes*, *Moniliales*, *Moniliaceae*, *Acremonium*	No clear products			Modified Ellman’s method	Wang et al. [35]
*Acremonium* sp. 2F09P03B	*Eumycota*, *Deuteromycotina*, *Hyphomycetes*, *Moniliales*, *Moniliaceae*, *Acremonium*	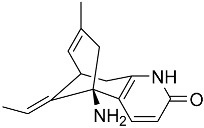	Huperzine A	0.6 μM	No report	Li et al. [37]
*Botrytis* sp. HA23	*Eumycota*, *Deuteromycotina*, *Hyphomycetes*, *Moniliales*, *Moniliaceae*, *Botrytis*	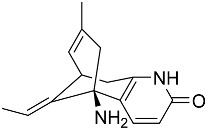	Huperzine A	0.6 μM	No report	Ju et al. [38]
*Trichoderma* sp.	*Eumycota*, *Deuteromycotina*, *Hyphomycetes*, *Moniliales*, *Moniliaceae*, *Trichoderma*	No clear products			Modified Ellman’s method	Dong et al. [39]
*Colletotrichum gloeosporioides* ES026	*Eumycota*, *Deuteromycotina*, *Coelomycetes*, *Melanconiales*, *Melanconiaceae Colletotrichum*	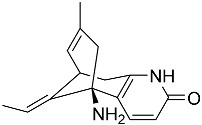	Huperzine A	0.6 μM	Modified Ellman’s method	Zhao et al. [40]
*Alternaria* sp. YD-01	*Eumycota*, *Deuteromycotina*, *Hyphomycetes*, *Moniliales*, *Dematiaceae Alternaria*	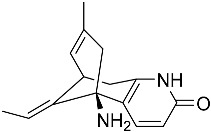	Huperzine A	0.6 μM	TLC bioautography	Yang et al. [41]
*Cladosporium cladosporioides* LF70	*Eumycota*, *Deuteromycotina*, *Hyphomycetes*, *Moniliales*, *Dermateaceae*, *Cladosporium*	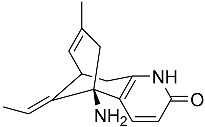	Huperzine A	0.6 μM		
*Phomopsis* sp.	*Eumycota*, *Deuteromycotina*, *Coelamycetes*, *Sphaeropsidales*, *Sphaeropsidaceae*, *Phomopsis*	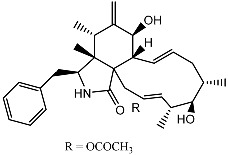	Cytochalasin H	No report	TLC bioautography	Chapla et al. [42]
*Chrysosporium* sp.	*Eumycota*, *Ascomycotina*, *Ascomycetes*, *Onygenales*, *Onygenaceae, Chrysosporium*	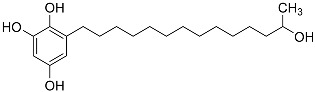	14-(2′,3′,5′-Trihydroxyphenyl)tetradecan-2-ol	197 μM	Modified Ellman’s method	Sekhar Rao et al. [43]
*Shiraia* sp. Slf14	*Eumycota*, *Ascomycotina*, *Pyrenomycetes*, *Sphaeriales*, *Hypocreaceae*, *Shiraia*	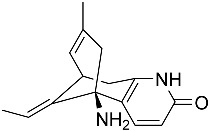	Huperzine A	0.6 μM	Modified Ellman’s method	Zhu et al. [44]
*Xylaria* sp. YS-02	*Eumycota*, *Ascomycota*, *pyrenomycetes*, *Sphaeriales*, *Xylariaceae*, *Xylaria*	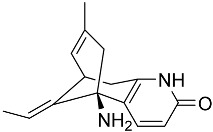	Huperzine A	0.6 μM	Modified Ellman’s method	Su et al. [45]
*Xylaria* sp.	*Eumycota*, *Ascomycota*, *pyrenomycetes*, *Sphaeriales*, *Xylariaceae*, *Xylaria*	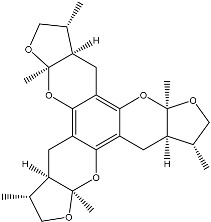	Xyloketal A	29.9 μM	Modified Ellman’s method	Lin et al. [46]
		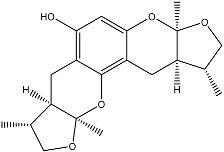	Xyloketal B	109.3 μM		
		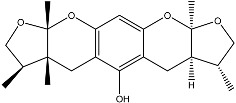	Xyloketal C	109.3 μM		
		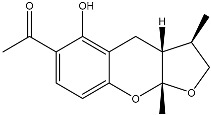	Xyloketal D	425.6 μM		
*Hyalodendriella* sp. Ponipodef12	*Eumycota*, *Ascomycotina*, *Discomycetes*, *Leotiales*, *Leotiaceae*, *Hyalodendriella*	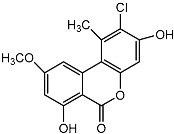	Palmariol B (**1**)	115.31 µg/mL	Modified Ellman’s method	Meng et al. [47], Mao et al. [48]
		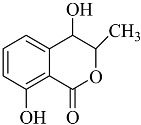	4-Hydroxymellein (**2**)	116.05 µg/mL		
		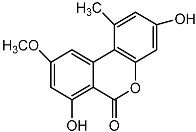	Alternariol 9-methyl ether (**3**)	135.52 µg/mL		
		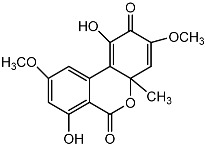	Botrallin (**4**)	83.70 µg/mL		
*Blastomyces* sp.	*Eumycota*, *Ascomycotina*, *Hemiascomycetes*, *Endomycetalcs*, *Endomycetaccae*, *Blastomyces*	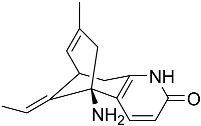	Huperzine A	0.6 μM	No report	Ju et al. [38]
*Haddowia longipes*	*Eumycota*, *Basidiomycotina*, *Hymenomycetes*, *Aphyllophorales*, *Ganodermataceae*, *Haddowia*	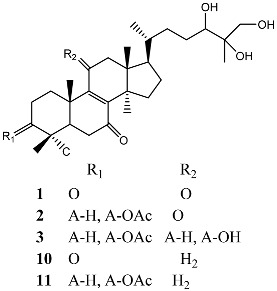	11-Oxo-ganoderiol D (**1**) Lanosta-8-en-7,11-dioxo-3b-acetyloxy-24,25,26-trihydroxy (**2**) Lanosta-8-en-7-oxo-3b-acetyloxy-11b,24,25,26-tetrahydroxy (**3**) Ganoderiol D (**10**) Lanosta-8-en-7-one-3b-acetyloxy-24,25,26-trihydroxy (**11**)	No report	Modified Ellman’s method	Zhang et al. [49]
		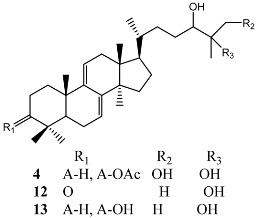	Lanosta-7,9(11)-dien-3b-acetyloxy-24,25,26-trihydroxy (**4**) Ganodermanondiol (**12**) Lucidumol B (**13**)			
		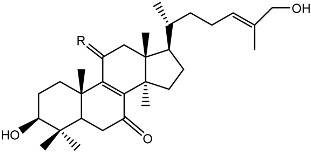	11β-Hydroxy-lucidadiol (**7**) Lucidadiol (**15**)			
		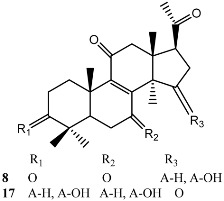	Lucidone H (**8**) lucidadone A (**17**)			
*Bacillus subtilis*	Bacteria, *Firmicutes*, *Bacilli*, *Bacillales, Bacillaceae*, *Bacillus*	No clear product			TLC bioautography and Modified Ellman’s method	Pandey et al.; Wang et al. [50,51]
*Streptomyces* sp. AH-14	*Phylum Actinobacteria*, *Actinomycetales*, *Streptomycetaceae*, *Streptomyces*	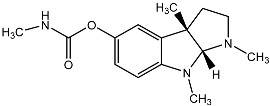	Physostigmine	41 μM	Modified Ellman’s method	Murao and Hayashi [52]
*Streptomyces* sp. LB173	*Phylum Actinobacteria*, *Actinomycetales*, *Streptomycetaceae*, *Streptomyces*	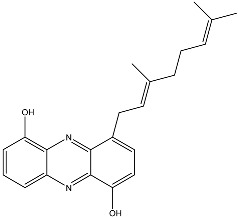	Geranylphenazinediol	2.62 μM	Modified Ellman’s method	Ohlendorf et al. [53]
*Streptomyces lavendulae*	*Phylum Actinobacteria*, *Actinomycetales*, *Streptomycetaceae*, *Streptomyces*	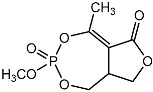	Oxygen heterocyclic compound	7.6 μM	Modified Ellman’s method	Kurokawa et al. [54]
*Rubrobacter radiotolerans*	*Phylum Actinobacteria*, *Rubrobacteridae*, *Rubrobacterales*, *Rubrobacteraceae, Rubrobacter*	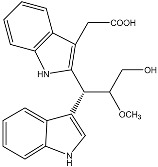	2-(2-(3-Hydroxy-1-(1*H*-indol-3-yl)-2-methoxypropyl)-1*H*-indol-3-yl)acetic acid	11.8 μM	Modified Ellman’s method	Li et al. [55]
		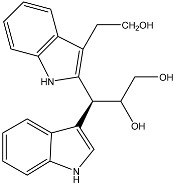	3-(3-(2-Hydroxyethyl)-1*H*-indol-2-yl)-3-(1Hindol-3-yl)propane-1,2-diol	13.5 μM		
N98-1021	*Phylum Actinobacteria*, *Actinomycetales*	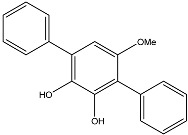	Same structure as terferol	20 μM	Modified Ellman’s method	Dong et al. [56]
*Streptosporangium* sp.	*Phylum Actinobacteria*, *Actinomycetales*, *Streptomycetaceae*, *Streptosporangium*	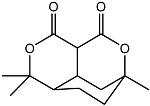	YXJ-E1 Novel compound extracted from secondary metabolites of *Actinobacteria* for the first time	No report	Modified Ellman’s method	Yang et al. [57]
		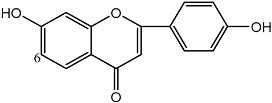	7,4′-Dihydroxy flavone	No report		
*Talaromyces* sp. LF458	*Phylum Actinobacteria*, *Actinomycetales*, *Arthrobacter Talaromyces*	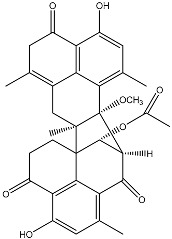	Talaromycesone A	7.49 μM	Modified Ellman’s method	Wu et al. [58]
*Cladonia macilenta Hoffm.*	Lichenes, *Ascolichens*, *Cladoniaceae*, *Cladonia*	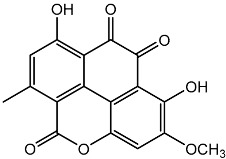	Biruloquinone 9,10-Phenanthrene quinone, a rare natural quinone compound	27.1 μg/mL	Modified Ellman’s method	Luo et al. [59]
Nostoc 78-12A	Cyanophyta, *Nostocales*, *Nostocaceae*, *Nostoc*	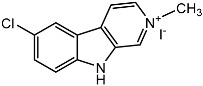	Nostocarboline	5.3 μM	Modified Ellman’s method	Becher et al. [60]
